# Bespoke and Accessible Electrochemical Reactors

**DOI:** 10.1021/acscentsci.4c01794

**Published:** 2024-11-20

**Authors:** Charlotte E. Willans

**Affiliations:** Department of Chemistry, University of York, Heslington, York, YO10 5DD, U.K.

Electrosynthesis, the use of electricity to drive chemical reactions, has the potential to revolutionize the chemicals industry.^1^ It avoids the use of harsh chemical reagents and allows a greater degree of control over reaction conditions compared to chemical reactions, which can lead to fewer byproducts (greater selectivity) and less waste (greater sustainability). In addition to the parameters that control traditional chemical reactions such as reagent concentration, catalyst, and stirring rate, electrochemical reactions are dependent upon several further factors such as electrode potential, current density, electrode material, and electrolyte concentration.^2,3^ Reaction cell design also has a huge impact on the outcome of electrochemical reactions, as the electron transfer process is heterogeneous. Therefore, features such as the configuration of the electrodes, the distance between them, reactor volume, mixing, and atmosphere (atmospheric or inert) will affect the efficiency and selectivity of electrochemical reactions. In this issue of *ACS Central Science*, a team lead by Alastair Lennox report user-friendly software that allows several electrochemical reactor parameters to be modified, thus enabling bespoke and reproducible reactors to be 3D printed.^4^

Electrosynthesis and electrocatalysis may be conducted in either an undivided or a divided cell,^5^ with both batch and flow options possible for both. Slightly more elaborate setups involving the use of a reference electrode are required for electroanalytical chemistry, which can be a powerful tool to aid mechanistic understanding of electrochemical reactions.^6^ As electrochemistry has undergone a renaissance within the synthetic community over the past decade, its progress has been supported by the development of commercial batch and flow reactors of different scales, in addition to high-throughput screening platforms.^7−10^ The adoption of reactor standardization within the electrochemical field is key to reproducibility and further development of the reported reactions.

**Figure 1:**
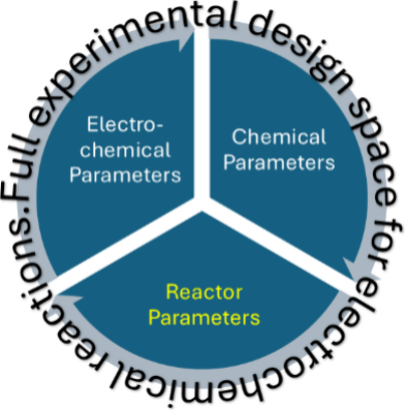
All the parameters that affect the outcome of electrochemical reactions.

This standardization does, however, fix one of the crucial parameters that can modify and help optimize electrochemical processes, that is, the reactor configuration. As Lennox and co-workers highlight in their manuscript, the heterogeneous reaction creates vessel-dependent effects on reaction outcome.^1^ Without the ability to easily vary reactor parameters in a standardized way, these effects cannot be reproducibly explored. This *ACS Central Science* article addresses these major limiting factors by developing software that enables the full experimental design space for electrochemical reactions to be explored (Figure 1).

Computer-aided design (CAD) software, of which there are many different packages, can be used to design objects such as reactor vessels. The output is often in the form of electronic files which can be used for printing or machining. Iterating upon designs is only possible with specific user training and understanding. Lennox and co-workers have developed a tool, which they have named ERCAD (Electrochemical Reactor Computer Aided Design), that sits between CAD and the user. A menu is provided of “chemistry-relevant” parameters that can be inputted using drop-down lists or scales to create bespoke electrochemical reactors that are easily reproducible across different labs.

**Figure 2:**
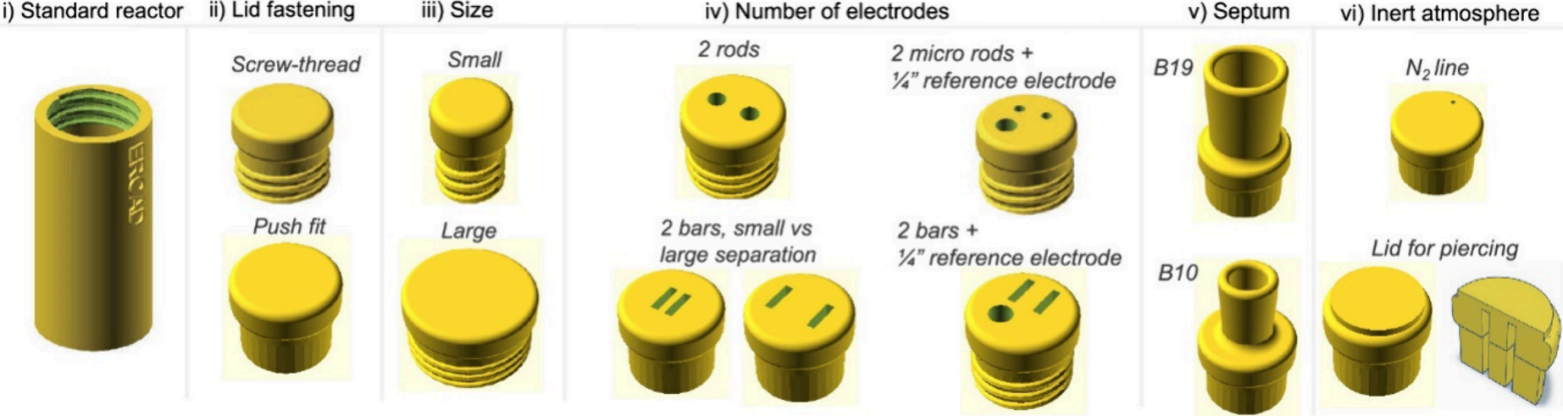
Selection of reactor lids that can be produced using ERCAD. Reproduced with permission from ref (4). Copyright 2024 American Chemical Society.

Reactors that are designed through ERCAD are produced in two pieces. The reactor vessel can be modified to varying volume, wall thickness, and base curvature. The lid has several variables such as number, relative position, and size of electrode holders, which allows parameters such as interelectrode gap and current density to be investigated (Figure 2). Both divided and undivided cells are accessible, and lids of different sizes are configured to be either screw thread or push-fit.

Using ERCAD, the group have benchmarked several different reactors, all of which have been 3D printed from polypropylene. A cyclic voltammogram of 1-*tert*-butyl-4-ethylbenzene was achieved using an 8 mL reactor fitted with a nitrogen needle and a 3-electrode configuration. An electrochemically promoted N-N-dimerization reaction was used to investigate the effect of electrode separation, through the production of different lids with electrode holders positioned at different distances apart. An electrochemical alkene difluorination reaction was conducted in a divided cell produced through ERCAD with good product yield. A 40 mL reactor vessel was used in combination with the ElectraSyn platform to scale-up an electrochemical acetoxylation, with a screening setup of eight 8 mL reactor vials being produced to explore substrate scope.

While several reactor types have been produced and tested in this early work, broad adoption of the promising design tool by electrochemists and electrosynthetic chemists will be essential to realize its full potential. Demonstrating reproducibility and systematic optimization across different laboratories will be necessary to truly test the standardization of this approach. The open access nature of the software, in addition to detailed instructions for installation and use within the supplementary information, should enable this to happen. The need for access to a 3D printer and printing materials may be an obstacle; however, these resources are becoming more commonplace and, arguably, should be standard equipment within laboratories.
